# Associations of Walking Activity With Hypertensive Mediated Organ Damage in Community-Dwelling Elderly Chinese: The Northern Shanghai Study

**DOI:** 10.3389/fcvm.2021.734766

**Published:** 2021-10-21

**Authors:** Yuyan Lyu, Shikai Yu, Chen Chi, Jiadela Teliewubai, Jue Li, Jacques Blacher, Jun Pu, Yi Zhang, Yawei Xu

**Affiliations:** ^1^Department of Cardiology, Renji Hospital, School of Medicine, Shanghai Jiao Tong University, Shanghai, China; ^2^Department of Cardiology, Shanghai Tenth People's Hospital, Tongji University School of Medicine, Shanghai, China; ^3^Department of Prevention, Tongji University School of Medicine, Shanghai, China; ^4^Key Laboratory of Arrhythmias, Ministry of Education, Tongji University School of Medicine, Shanghai, China; ^5^Department of Therapeutics, Paris-Descartes University, Paris, France; ^6^AP-HP, Paris, France; ^7^Diagnosis and Therapeutic Center, Hôtel-Dieu, Paris, France

**Keywords:** walking activity, cardiovascular disease risk factors, hypertensive mediated organ damage, elderly, vascular protection

## Abstract

**Background:** Walking, as the most common campaign in older people, is recommended to improve their cardiovascular health. However, the direct association between weekly walking activity and asymptomatic hypertensive mediated organ damage (HMOD) remains unclear.

**Methods:** 2,830 community-dwelling elderly subjects (over 65 years) in northern Shanghai were recruited from 2014 to 2018. Weekly walking activity was assessed by International Physical Activity Questionnaires (IPAQ). Within the framework of comprehensive cardiovascular examinations, HMOD, including left ventricular mass index, peak transmitral pulsed Doppler velocity/early diastolic tissue Doppler velocity, creatinine clearance rate, urinary albumin–creatinine ratio, carotid-femoral pulse wave velocity (cf-PWV), carotid intima–media thickness (CIMT), arterial plaque, and ankle-brachial index (ABI), were all evaluated.

**Results:** 1,862 (65.8%) participants with weekly walking activity showed lower CIMT, lower cf-PWV, fewer abnormal ABI, and lower prevalence of hypertension and coronary heart disease (*p* < 0.05). Walking activity was negatively correlated with age and smokers (correlation coefficient: −0.066, −0.042; both *p* < 0.05). After adjusting for cardiovascular risk factors and concomitant diseases, walking activity was significantly associated with better indicator of most vascular HMOD in multivariate logistic regressions, including arterial stiffness [odds ratio (OR) = 0.75, *p* = 0.01], increased CIMT (OR = 0.70, *p* = 0.03), and peripheral artery disease (OR = 0.72, *p* = 0.005), but not cardiac or renal HMOD. Subgroup analysis further showed that walking duration ≥1 h/day was significantly associated with decreased risk of most vascular HMOD after adjustment for confounders and moderate-to-vigorous physical activity based on IPAQ (all *p* < 0.05).

**Conclusions:** In the community-dwelling elderly Chinese, there was a significant negative association of weekly walking activity with vascular HMOD, but not cardiac or renal HMOD. Increased daily walking duration, but not walking frequency, was significantly associated with improved vascular HMOD. Hence, increasing daily walking duration seems to encourage a healthy lifestyle in terms of vascular protection.

**Clinical Trial Registration:** ClinicalTrials.gov, identifier: NCT02368938.

## Introduction

Epidemiological studies in China showed a rapidly increasing prevalence of hypertension from 18.0% in 2002 to 27.8% in 2014 (1). Although awareness and treatment of hypertension were improved, the control rate of hypertension remains extremely low, resulting in the increasing mortality and huge economic burden to the society. Effective therapies to manage hypertension and hypertensive mediated organ damage (HMOD) are needed at a population level, especially in the elderly. Notably, besides treatment with anti-hypertensive medicine, physical activity also plays an important role in the prevention and control of elevated blood pressure, such as walking, morning exercise, square dancing, Yoga, etc. (2). Therefore, physical activity could be a potential nature therapy for many cardiovascular diseases (CVDs).

Accumulating evidence demonstrated that physical activity (including low, moderate, and vigorous intensity) could prevent CVD, exhibit multi-system anti-aging effects (3), and extend the life expectancy of the world's population, partly due to the improvement in cardiovascular (CV) risk factors (4, 5). The American College of Sports Medicine (ACSM) recommends that older adults do moderate intensity cardiorespiratory physical activity training for 30 min/day on 5 days/week, vigorous intensity training for 20 min/day on 3 days/week, or a combination of moderate and vigorous intensity training to consume up to 500–1,000 MET-minutes/week of energy (6). Similarly, WHO recommends more than 150 min/week of moderate intensity or more than 75 min/week of vigorous intensity physical activity, or an equivalent combination of moderate and vigorous intensity physical activity (version 2010) (7). However, the level of physical activity among the elderly tends to decrease along with the increase of age. Hence, elderly with hypertension presents a challenge to achieve the recommended physical activity, especially moderate or vigorous intensity physical activity (8). Walking as a low level of physical activity is easily to be accepted in the elderly. Walking involves the interaction of neuromuscular, sensory, and cognitive functions without a high risk of injury, great difficulties, additional cost, or exercise equipment (9). Numerous studies have demonstrated the beneficial effects of walking on cardiovascular protection (10–13); nevertheless, there are no detailed guidelines on the suggested walking time and weekly frequency for the elderly. It is noteworthy that walking over 60 min/week at a leisurely pace did not improve cardiorespiratory fitness (14). Furthermore, Foster et al. in the Framingham Heart Study found no association between physical activity with indices of kidney function over an average follow-up of 6.6 years (15). Thus, it is controversial whether weekly walking activity influenced the HMOD. Considering asymptomatic HMOD in the elderly as critical prodromes of CV events and mortality, we investigated the association of weekly walking activity with asymptomatic HMOD (cardiac HMOD, renal HMOD, and vascular HMOD) based on self-reported walking participation within the framework of CV risk assessment in a community-dwelling elderly.

## Methods

### Study Design

The Northern Shanghai Study is a prospective, ongoing, and multistage study, and aims to investigate the CV risk assessment system in the elderly Chinese, as previously described (16, 17). We recruited residents from urban communities in the north of Shanghai (aged 65 years or more), who are also available for long-term follow-up. Subjects with severe cardiac disease (NYHA IV) or end-stage renal disease (CKD > 4), or malignant tumor with life expectancy <5 years, or stroke history within 3 months were excluded. Finally, 2,830 participants were enrolled from June 2014 to May 2018, including 1,259 (44.5%) male, 722 (25.5%) smokers, 1,530 (54.1%) with hypertension, 566 (20.0%) with diabetes mellitus, and 937 (33.1%) with coronary heart disease. The study was approved by the Ethics Committee of Shanghai Tenth People's Hospital, and written informed consent was obtained from each participant. Of note, the current study is a cross-sectional study from the Northern Shanghai Study to investigate the direct association between weekly walking activity and asymptomatic HMOD.

### Definition of Weekly Walking Activity

Weekly walking activity was evaluated by standard questionnaires based on the International Physical Activity Questionnaires-short form (IPAQ, including how many days spent on walking at least 10 min at a time and walking duration time) (http://www.ipaq.ki.se), which has been validated and widely used in many clinical trials (18–20). In subgroup analysis, walking duration was classified into four groups (non-walking activity; 10–29 min/day; 30–59 min/day; ≥1 h/day), and walking frequency were categorized into <3 days/week and ≥3 days/week, with reference to the Korean National Health and Nutrition Examination Surveys (KNHANES) (18).

Furthermore, the metabolic equivalent of tasks (METs) was calculated as follows: walking activity METs = 3.3 × walking minutes × walking days (20). Then, physical activity was divided into three levels (low, moderate, vigorous intensity) as previously reported: vigorous, seven or more days of walking achieving at least 3,000 MET-minutes/week; moderate, five or more days of walking achieving at least 600 MET-minutes/week; low, not meeting the criteria of moderate or vigorous intensity (20, 21).

### Social, Clinical, and Biological Parameters

We obtained social and clinical information from standard questionnaires, including gender, age, body weight, body height, smoking habits, history of hypertension/diabetes mellitus/coronary heart disease, etc. (16).

As to biological markers, venous blood samples and urine samples were obtained from subjects after an overnight fast. Biological markers were measured in the Department of Laboratory Medicine of Shanghai Tenth People's Hospital, including plasma creatinine (PCr), urinary microalbumin and creatinine, etc. We calculated creatinine clearance rate (CCR) and urinary albumin–creatinine ratio (UACR) based on the modified MDRD formula for Chinese and urinary microalbumin divided by urinary creatinine, respectively (16, 17).

### Measurement of Blood Pressure, Ankle-Brachial Index, and Carotid-Femoral Pulse Wave Velocity

Specialized physicians measured the blood pressure (BP) of each subject in the morning by the electronic device three times after at least 10 min of rest in the sitting position, according to the recommendations of the European Society of Hypertension (22). The average of three BP readings was used in the subsequent statistical analysis.

Bilateral brachial and ankle blood pressures were measured and ankle-brachial index (ABI, calculated as ankle systolic BP divided by brachial systolic BP) was automatically calculated via the VP1000 system (Omron, Japan), according to the recommendations of the American Heart Association (23). Lower ABI was used for analysis in the present study.

Carotid-femoral pulse wave velocity (Cf-PWV) as a non-invasive golden standard was recommended to assess the arterial stiffness (Class I, Level of Evidence A) using SphygmoCor system (AtCor Medical, Australia) (24, 25). Briefly, after a 10-min rest, peripheral BP was recorded twice with an interval of 3 min, and measurements of the superficial distance directly from the carotid to the femoral artery were performed. Subsequently, pressure waveforms in the right carotid and right femoral arteries were recorded, and transit time for each artery was automatically calculated *via* ECG data. Finally, cf-PWV was calculated by traveling distance divided by traveling time. Notably, an operator index > 80% indicated a high-quality waveform.

### Ultrasonography

All ultrasonographic measurements were performed by a single experienced sonographer. Arterial plaque and common carotid artery intima–media thickness (CIMT) was assayed by the MyLab 30 Gold CV system (ESAOTE SpA, Genoa, Italy). The presence or absence of plaques in the left and right carotid arteries was recorded. Also, CIMT was measured on the left common carotid artery (always on plaque-free arterial segments), 2 cm from the bifurcation, as previously described (16, 17). The average value of three CIMT measurements was used for further analysis.

Furthermore, M-mode and 2-dimensional echocardiography were performed using the same device, according to the guidelines of the American Society of Echocardiography (ASE) (16, 17). From the parasternal view, we measured left ventricular end-diastolic diameter (LVEDd), interventricular septal (IVSd) and posterior wall thickness at end-diastole (PWTd), and then calculated left ventricular mass index (LVMI) as previously described (18, 19). Simultaneously, peak transmitral pulsed Doppler velocity/early diastolic tissue Doppler velocity (E/Ea) was calculated for the evaluation of LV diastolic function. In addition, left atrial volume index (LAVI) was calculated using model formula as previously described (18, 19).

### Definition of Asymptomatic Hypertensive Mediated Organ Damage

Asymptomatic HMOD included cardiac, renal, and vascular HMOD. With regard to cardiac HMOD, left ventricular hypertrophy was defined as LVMI ≥ 115 g/m^2^ (male) or LVMI ≥ 95 g/m^2^ (female) (26), and LV diastolic dysfunction was defined as E/Ea ≥ 15, or 15 > E/Ea > 8 with any of the following: LAVI > 40 ml/m^2^ or LVMI > 149 g/m^2^ (male) or LVMI ≥ 122 g/m^2^ (female) (27). Chronic kidney diseases (CCR <60 ml/min/1.73 m^2^) and microalbuminuria (UACR > 30) represented renal HMOD (28), while vascular HMOD included the presence of arterial plaque, increased CIMT (CIMT > 900 μm), arterial stiffness (cf-PWV ≥ 12 m/s), and peripheral artery disease (ABI < 0.9) (16, 17, 29).

### Statistical Analysis

Data are presented as means ± SD or frequencies (percentage). Continuous variables were compared by unpaired Student's *t*-test for normally distributed variables or the Mann–Whitney *U* test when variables were not normally distributed. For multiple comparisons, the ANOVA test was conducted using Duncan's multiple range test to investigate the association of walking duration with vascular HMOD. Comparison of categorical variables was evaluated by χ^2^ test. Pearson's correlation analysis was applied to investigate the correlation of CV risk factors with weekly walking activity. The odds ratio (OR) and 95% CI of weekly walking activity were calculated for the risk of HMOD. Multivariate logistic regressions were performed to investigate the association of walking activity with HMOD, together with CV risk factors (including age, gender, smokers, body mass index, systolic blood pressure) and concomitant diseases (hypertension, diabetes mellitus, coronary heart disease). In subgroup analysis, we conducted multivariate logistic regressions to assess the relationship between risk of vascular HMOD and walking duration/frequency/different levels of physical activity. Statistical analysis was performed using SAS software, version 9.3 (SAS Institute, Cary, NC, USA). *P* < 0.05 was considered statistically significant.

## Results

### Characteristics of Study Participants

Detailed characteristics of participants are presented in Table 1, including CV risk factors, asymptomatic HMOD, and concomitant diseases. There were 1,862 (65.8%) participants enrolled in weekly walking activity. Participants with walking activity, compared with non-walking activity, were younger and had fewer smokers (both *p* < 0.05). Interestingly, there was no significant difference between participants with and without walking activity in cardiac and renal HMOD (all *p* > 0.05). As to vascular HMOD, participants with walking activity had significantly lower cf-PWV (*p* = 0.004), lower CIMT (*p* = 0.001), and lower percentage of participants with abnormal ABI (*p* = 0.003). In addition, in comparison with non-walking activity group, participants with walking activity had a lower percentage of concomitant diseases, such as hypertension (*p* = 0.006) and coronary heart disease (*p* = 0.008).

**Table 1:** Characteristics of participants by walking activity.

	**Overall (*n* = 2,830)**	**Walking activity (*n* = 1,862)**	**Non-walking activity (*n* = 968)**	***P***
**Cardiovascular risk factors**				
Age (years)	71.53 ± 6.25	71.23 ± 6.04	72.10 ± 6.61	** <0.001**
Male gender, *n* (%)	1,259 (44.5)	836 (44.9)	423 (43.7)	0.54
Smokers, *n* (%)	722 (25.5)	451 (24.2)	271 (28.0)	**0.03**
Body weight (kg)	62.69 ± 0.80	62.61 ± 10.51	62.84 ± 11.34	0.60
Body height (cm)	159.8 ± 8.4	159.9 ± 8.3	159.7 ± 8.7	0.52
Body mass index (kg/m^2^)	24.02 ± 3.64	23.97 ± 3.54	24.11 ± 3.83	0.35
Systolic blood pressure (mmHg)	135.1 ± 17.4	134.7 ± 17.4	135.8 ± 17.4	0.09
**Hypertensive mediated organ damage**				
Left ventricular mass index (g/m^2^)	90.4 ± 28.9	90.4 ± 28.2	90.5 ± 30.2	0.92
E/Ea	9.67 ± 3.91	9.59 ± 3.83	9.82 ± 4.05	0.18
Creatinine clearance rate (ml/min/1.73 m^2^)	93.2 ± 24.3	93.7 ± 24.7	92.3 ± 23.5	0.65
Urinary albumin–creatinine ratio (mg/g)	65.3 ± 153.5	64.5 ± 172.5	67.1 ± 107.6	0.65
Carotid-femoral pulse wave velocity (m/s)	9.49 ± 2.33	9.39 ± 2.23	9.67 ± 2.50	**0.004**
CIMT (μm)	638.1 ± 159.1	631.1 ± 156.8	650.8 ± 162.8	**0.001**
Arterial plaque, *n* (%)	1,780 (63.6)	1,166 (63.1)	614 (63.4)	0.48
Ankle-brachial index <0.9, *n* (%)	358 (13.5)	212 (12.1)	146 (15.1)	**0.003**
**Concomitant diseases**				
Hypertension, *n* (%)	1,530 (54.1)	973 (52.3)	557 (57.5)	**0.006**
Diabetes mellitus, *n* (%)	566 (20.0)	381 (20.5)	185 (19.1)	0.41
Coronary heart disease, *n* (%)	937 (33.1)	586 (31.5)	351 (36.3)	**0.008**

*Bold values indicate significant results (P < 0.05). Walking activity was defined as walking more than 10 min at a time per week. Data are means ± SD or numbers with percentages in parentheses. Student's *t*-test (or Mann–Whitney *U* test) and χ^2^ test were conducted to compare the differences between walking activity and non-walking activity groups for quantitative and qualitative variables, respectively. Creatinine clearance rate was calculated with modified MDRD formula for Chinese. CIMT, carotid intima–media thickness; E/Ea, peak transmitral pulsed Doppler velocity/early diastolic tissue Doppler velocity*.

### Correlation of Cardiovascular Risk Factor With Walking Activity

Correlation analysis was performed to investigate the correlation of CV risk factors with weekly walking activity. In line with the results in Table 1, walking activity was only correlated with age and smokers (both *p* < 0.05), but not male gender, body weight, body height, body mass index, or systolic blood pressure (all *p* > 0.05) (Supplementary Table 1).

### Association of Weekly Walking Activity With Asymptomatic HMOD

**Figure 1:**
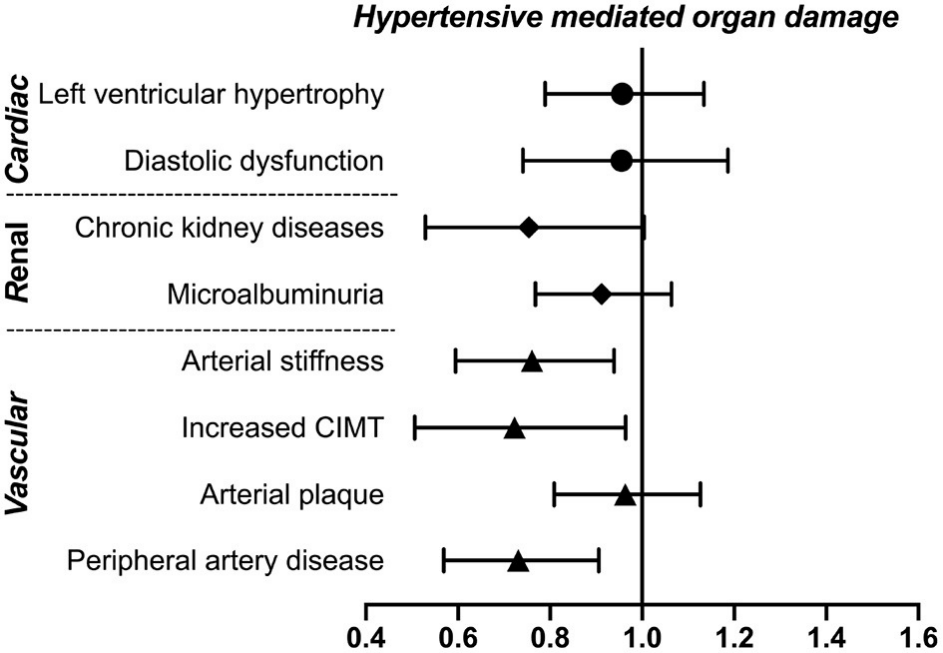
Association of weekly walking activity with asymptomatic HMOD. Weekly walking activity was defined as walking more than 10 min at a time per week. The ORs and 95% CI of walking activity were presented after adjustment for cardiovascular risk factors (including age, gender, smokers, body mass index, systolic blood pressure) and concomitant diseases (hypertension, diabetes mellitus, coronary heart disease) using multivariate logistic regressions. CIMT, carotid intima–media thickness; HMOD, hypertensive mediated organ damage; OR, odds ratio.

To investigate the association of weekly walking activity with asymptomatic HMOD, multivariate logistic regressions were conducted, together with CV risk factors and concomitant diseases. As demonstrated in Figure 1, weekly walking activity is significantly associated with a low risk of most vascular HMOD, including arterial stiffness (OR = 0.75, 95% CI: 0.60–0.94, *p* = 0.01), increased CIMT (OR = 0.70, 95% CI: 0.51–0.96, *p* = 0.03), and peripheral artery disease (OR = 0.72, 95% CI: 0.57–0.91, *p* = 0.005), but not cardiac or renal HMOD (all *p* > 0.05).

### Association of Walking Duration and Frequency With Vascular HMOD

**Figure 2:**
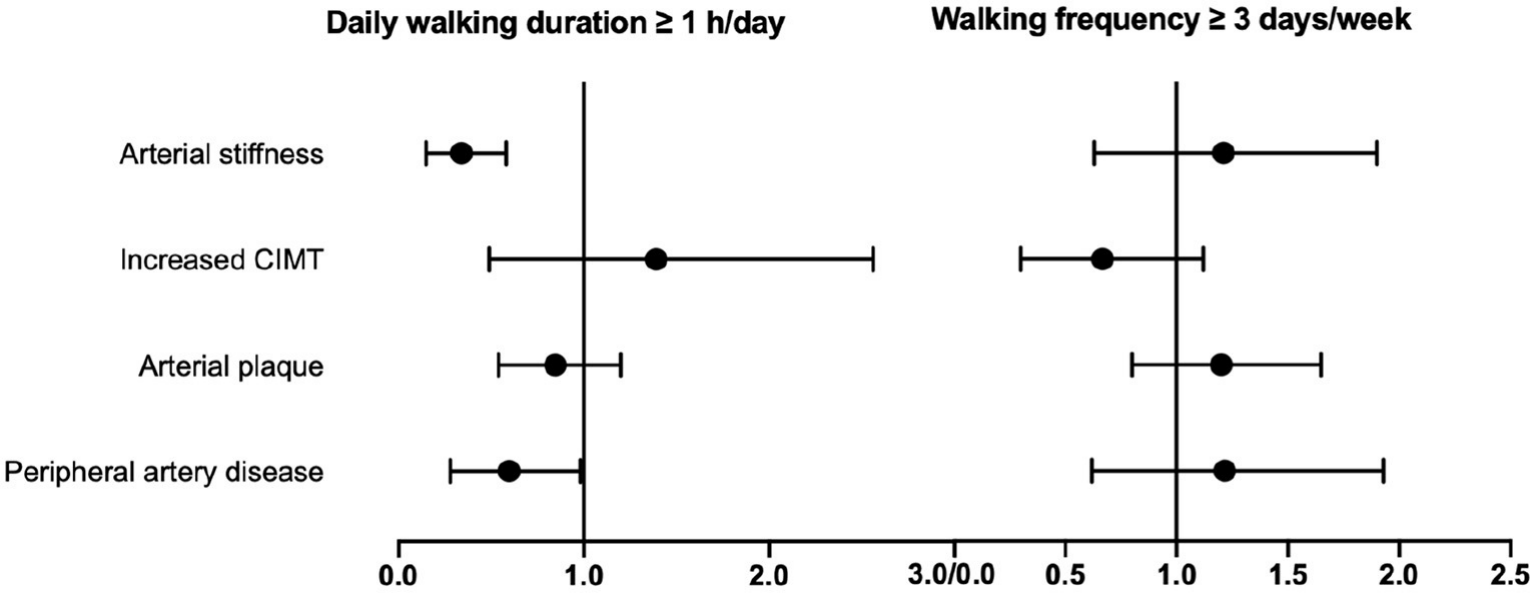
Subgroup analysis of the association of daily walking duration or walking frequency with vascular HMOD. Multivariate logistic regressions were performed to investigate the association of walking duration ≥1 h/day or walking frequency ≥ 3 days/week with vascular HMOD. Adjusted ORs and 95% CI are presented after adjustment for walking frequency or walking duration, together with cardiovascular risk factors (including age, gender, smokers, body mass index, systolic blood pressure) and concomitant diseases (hypertension, diabetes mellitus, coronary heart disease), respectively. CIMT, carotid intima–media thickness; HMOD, hypertensive mediated organ damage; OR, odds ratio.

Next, we performed subgroup analysis to investigate the association of walking duration and frequency with vascular HMOD. First, all participants were divided into four groups according to walking duration as previously described (18), including non-walking activity, 10–29 min/day, 30–59 min/day, and ≥1 h/day groups. Along with the increase of walking duration, cf-PWV, CIMT, and ABI were significantly and gradually improved, as well as the decreased prevalence of arterial stiffness, increased CIMT, and peripheral artery disease (all *p* < 0.05) based on Duncan's multiple range tests (Supplementary Table 2). Moreover, multivariate logistic regressions were performed to validate the risk of vascular HMOD across different walking duration without and with adjustment for walking days per week, together with risk factors and concomitant diseases. Walking duration ≥1 h/day was closely associated with lower crude ORs and adjusted ORs for most vascular HMOD than non-walking activity (*p* < 0.05) (Table 2; Figure 2).

**Table 2:** Risk of vascular HMOD across different walking duration.

**Vascular HMOD**		**Crude OR (95% CI)**	**Adjusted OR (95% CI)**
Arterial stiffness (*n* = 2,723)	Non-walking activity	1	1
	≥10–29 min/day	0.75 (0.41–1.37)	**0.38 (0.17–0.85)**
	≥30–59 min/day	0.84 (0.63–1.10)	**0.38 (0.20–0.74)**
	≥1 h/day	**0.67 (0.52–0.87)**	**0.29 (0.15–0.58)**
Increased CIMT (*n* = 2,810)	Non-walking activity	1	1
	≥10–29 min/day	0.74 (0.31–1.74)	0.94 (0.33–2.66)
	≥30–59 min/day	0.82 (0.58–1.17)	0.68 (0.30–1.56)
	≥1 h/day	**0.52 (0.33–0.81)**	1.12 (0.49–2.56)
Arterial plaque (*n* = 2,807)	Non-walking activity	1	1
	≥10–29 min/day	0.93 (0.62–1.41)	0.78 (0.47–1.30)
	≥30–59 min/day	0.89 (0.73–1.09)	0.73 (0.49–1.08)
	≥1 h/day	1.00 (0.83–1.20)	0.80 (0.54–1.20)
Peripheral artery disease (*n* = 2,663)	Non-walking activity	1	1
	≥10–29 min/day	0.81 (0.45–1.46)	0.69 (0.33–1.46)
	≥30–59 min/day	0.78 (0.59–1.03)	0.65 (0.35–1.18)
	≥1 h/day	**0.65 (0.50–0.84)**	**0.53 (0.28–0.99)**

*Bold values indicate significant results (P < 0.05). Multivariate logistic regressions were performed to investigate the risk of vascular HMOD across different walking duration without and with adjustment for walking days per week, together with cardiovascular risk factors (including age, gender, smokers, body mass index, systolic blood pressure) and concomitant diseases (hypertension, diabetes mellitus, coronary heart disease). CIMT, carotid intima–media thickness; HMOD, hypertensive mediated organ damage; OR, odds ratio*.

Then, walking frequency was categorized into walking <3 days/week and ≥3 days/week, and we found significant differences between the two groups in the prevalence of arterial stiffness, increased CIMT, and peripheral artery disease (all *p* < 0.05), except arterial plaque (*p* = 0.93) (Supplementary Table 3). Further, multivariate logistic regressions were conducted to confirm the risk of vascular HMOD across different walking frequency without and with adjustment for walking duration. The crude ORs of walking ≥ 3 days/week relative to <3 days/week were 0.79 (0.63–0.98), 0.65 (0.48–0.89), and 0.73 (0.58–0.91) for vascular HMOD (arterial stiffness, increased CIMT, peripheral artery disease), respectively (Table 3). Of note, after adjustment for walking duration, there was no association between walking frequency and vascular HMOD (all *p* > 0.05) (Table 3; Figure 2).

**Table 3:** Risk of vascular HMOD across different walking frequency.

**Vascular HMOD**		**Crude OR (95% CI)**	**Adjusted OR (95% CI)**
Arterial stiffness (*n* = 2,723)	<3 days/week	1	1
	≥3 days/week	**0.79 (0.63–0.98)**	1.10 (0.63–1.90)
Increased CIMT (*n* = 2,810)	<3 days/week	1	1
	≥3 days/week	**0.65 (0.48–0.89)**	0.58 (0.30–1.12)
Arterial plaque (*n* = 2,807)	<3 days/week	1	1
	≥3 days/week	0.97 (0.83–1.14)	1.15 (0.80–1.65)
Peripheral artery disease (*n* = 2,663)	<3 days/week	1	1
	≥3 days/week	**0.73 (0.58−0.91)**	1.10 (0.62–1.93)

*Bold values indicate significant results (P < 0.05). Multivariate logistic regressions were performed to investigate the risk of vascular HMOD across different walking days per week without and with adjustment for walking duration, together with cardiovascular risk factors (including age, gender, smokers, body mass index, systolic blood pressure), and concomitant diseases (hypertension, diabetes mellitus, coronary heart disease). CIMT, carotid intima–media thickness; HMOD, hypertensive mediated organ damage; OR, odds ratio*.

### Association of Walking Activity With Vascular HMOD Using Different Cut-Off Walking Duration

Afterwards, we used different cut-off walking duration per time (≥30 min/day or ≥1 h/day) to further confirm the association of walking frequency with vascular HMOD. As shown in Supplementary Table 4, under different cut-off walking duration, the prevalence of vascular HMOD, except arterial plaque, are consistently decreased between walking <3 and ≥3 days/week (*p* < 0.05). Subsequently, we checked the association of walking activity using different cut-off walking duration with vascular HMOD through multivariate logistic regressions. Consistent with aforementioned results using walking duration ≥10 min/day, weekly walking activity was also significantly associated with vascular HMOD using walking duration ≥30 min/day and ≥1 h/day (≥30 min/day: arterial stiffness, OR = 0.61, 95% CI: 0.77–0.96, *p* = 0.02; peripheral artery disease, OR = 0.73, 95% CI: 0.58–0.91, *p* = 0.006; ≥1 h/day: arterial stiffness, OR = 0.74, 95% CI: 0.58–0.94, *p* = 0.01; increased CIMT, OR = 0.72, 95% CI: 0.53–0.99, *p* = 0.04; peripheral artery disease, OR = 0.74, 95% CI: 0.58–0.94, *p* = 0.02).

### Association Between Different Levels of Physical Activity and Vascular HMOD

Furthermore, we calculated the METs and categorized into three levels of physical activity based on IPAQ (20), including low (*n* = 1,379), moderate (*n* = 1,411), and vigorous (*n* = 40). To investigate the relationship between different levels of physical activity and vascular HMOD, we conducted multivariate logistic regressions after adjustment for confounders, and the results indicated that only the resulting OR for peripheral artery disease was significantly negatively associated with a moderate-to-vigorous level of physical activity (OR: 0.74, 95% CI: 0.60–0.93, *p* = 0.009) relative to a low level of physical activity (Table 4). Finally, to enhance the association between walking duration/frequency and vascular HMOD, multivariate logistic regressions were conducted after adjusting for moderate-to-vigorous physical activity and similar results were observed (Table 5). These findings validated the association of increased daily walking duration with improved vascular HMOD.

**Table 4:** Risk of vascular HMOD across different levels of physical activity.

**Vascular HMOD**		**OR (95% CI)**
Arterial stiffness (*n* = 2,723)	Low	1
	Moderate to vigorous	0.90 (0.72–1.12)
Increased CIMT (*n* = 2,810)	Low	1
	Moderate to vigorous	0.73 (0.54–1.01)
Arterial plaque (*n* = 2,807)	Low	1
	Moderate to vigorous	1.06 (0.91–1.23)
Peripheral artery disease (*n* = 2,663)	Low	1
	Moderate to vigorous	**0.74 (0.60–0.93)**

*Bold values indicate significant results (P < 0.05). Multivariate logistic regressions were performed to investigate the risk of vascular HMOD across different levels of physical activity, together with cardiovascular risk factors (including age, gender, smokers, body mass index, systolic blood pressure) and concomitant diseases (hypertension, diabetes mellitus, coronary heart disease). CIMT, carotid intima–media thickness; HMOD, hypertensive mediated organ damage; OR, odds ratio*.

**Table 5:** Risk of vascular HMOD across different walking duration and frequency after adjustment for moderate-to-vigorous physical activity.

**Vascular HMOD**	**Walking duration**	**OR (95% CI)**	**Frequency**	**OR (95% CI)**
Arterial stiffness (*n* = 2,723)	Non-walking activity	1	<3 days/week	1
	≥10–29 min/day	0.35 (0.11–1.08)		
	≥30–59 min/day	**0.37 (0.18–0.75)**	≥3 days/week	0.80 (0.41–1.53)
	≥1 h/day	**0.29 (0.14–0.58)**		
Increased CIMT (*n* = 2,810)	Non-walking activity	1	<3 days/week	1
	≥10–29 min/day	0.80 (0.19–3.37)		
	≥30–59 min/day	1.06 (0.44–2.57)	≥3 days/week	0.54 (0.23–1.24)
	≥1 h/day	0.64 (0.26–1.58)		
Arterial plaque (*n* = 2,807)	Non-walking activity	1	<3 days/week	1
	≥10–29 min/day	1.07 (0.53–2.15)		
	≥30–59 min/day	0.82 (0.53–1.26)	≥3 days/week	0.98 (0.65–1.49)
	≥1 h/day	0.88 (0.58–1.36)		
Peripheral artery disease (*n* = 2,663)	Non-walking activity	1	<3 days/week	1
	≥10–29 min/day	0.41 (0.14–1.22)		
	≥30–59 min/day	0.54 (0.28–1.04)	≥3 days/week	1.14 (0.60–2.17)
	≥1 h/day	**0.48 (0.24–0.89)**		

*Bold values indicate significant results (P < 0.05). Multivariate logistic regressions were performed to investigate the risk of vascular HMOD across different walking duration and frequency after adjustment for moderate-to-vigorous physical activity, together with cardiovascular risk factors (including age, gender, smokers, body mass index, systolic blood pressure) and concomitant diseases (hypertension, diabetes mellitus, coronary heart disease). CIMT, carotid intima–media thickness; HMOD, hypertensive mediated organ damage; OR, odds ratio*.

## Discussion

The present study had two major findings. First, weekly walking activity was significantly associated with a lower risk of vascular HMOD, but not cardiac or renal HMOD. Second, subgroup analysis indicated that there was a significant association between increasing daily walking duration (≥1 h/day) and improved vascular HMOD in community-dwelling elderly population.

Accumulating evidence revealed that physical activity has an anti-aging effects in multi-system and increase the life expectancy (3). Both ACSM and WHO recommend adults engaging in moderate or vigorous intensity physical activity, or an equivalent combination (6, 7). Unlike moderate and vigorous intensity physical activity in elderly could potentially increase the risk of mortality, walking as a low intensity physical activity with a lower risk of injury than running or sport participation is easily accessible without any requirements of special equipment or training (30). Numerous studies have reported that walking exerts beneficial effects on reduction of CV risk, blood pressure, exercise capacity, cardiac capacity, maximal oxygen consumption, and quality of life in hypertensive patients with concomitant diseases (31–35), which is recommended to a wide range of people as primary and secondary prevention. Nevertheless, in our present study, walking activity was only significantly correlated with age and smokers, and no significant correlation existed between walking activity and body height, body weight, body mass index, or systolic blood pressure. The inconsistent findings might be partly due to a different level of physical activity and inclusive criteria. Thus, more randomized clinical trials are warranted to validate the correlation of walking activity and CV risk factors in the future.

In literature, physical activity is associated with adverse clinical outcomes of CVD (36, 37). However, it was still unclear whether weekly walking activity was associated with asymptomatic HMOD in an elderly population. First, few studies were conducted to investigate the relationship of walking activity with asymptomatic cardiac HMOD. Notably, a meta-analysis of prospective studies in 2021 showed that walking were not significantly associated with heart failure, although total physical activity, leisure-time activity, and vigorous activity were associated with a statistically significant decrease in the risk of heart failure (10). Similarly, there was no evidence of any association of self-reported walking activity on cardiac HMOD in our study, including left ventricular hypertrophy and LV diastolic dysfunction. Regarding the relationship between physical activity and renal HMOD, conflicting findings indeed existed. Some studies suggested that a high level of physical activity was associated with higher CCR and lower risk of CCR decline (38–40), while others showed no relationship between physical activity with CCR or UACR (15, 41–44). In this community-based elderly population, no association was observed between weekly walking activity and renal HMOD. With regard to vascular HMOD, several population-based studies indicated that physical activity was favorably associated with arterial stiffness (45) and atherosclerosis (46). In line with these results, our data indicated a significant association of walking activity on reduction of most vascular HMOD (including arterial stiffness, increased CIMT, and peripheral artery disease). Taken together, these findings suggested that vascular function and structure seemed to be influenced more than cardiac/renal abnormalities during walking activity in our community-based elderly population. Additional studies are needed to determine whether weekly walking activity prevent the onset or progression of vascular HMOD.

Accordingly, encouraging elderly subjects to walk could enable them to exercise at a low level against vascular HMOD. However, the required daily walking duration and walking frequency based on IPAQ remain unclear. Results of subgroup analysis showed a significant association of daily walking duration (especially ≥ 1 h/day) with most vascular HMOD after adjustment for walking frequency and all confounders. Intriguingly, there was no significant association of walking frequency with vascular HMOD after adjustment for walking duration and confounders. These outcomes suggested that daily walking duration ≥ 1 h/day was recommended to protect the elderly against vascular HMOD, but not walking frequency ≥ 3 days/week. To the best of our knowledge, this is the first study to investigate the relationship of self-reported weekly walking activity and asymptomatic HMOD according to daily walking duration and frequency of weekly walking activity in the elderly population. We indicated that, from the viewpoint of organ-protection-driven physical activity management, weekly walking activity (daily walking duration ≥ 1 h/day) was recommended for the Chinese elderly, especially those suffering from the vascular abnormalities.

### Limitations

Our results should be interpreted within the limitations. First, this study was a cross-sectional study using data from the Northern Shanghai Study. Therefore, it is difficult to derive causal relationship between weekly walking activity and HMOD. With ongoing follow-up studies, more accurate data will be provided in the future. Second, weekly walking activity was evaluated based on self-reported questionnaires (leisure time). The data were subject to error and potentially systematic bias. More studies will be required through targeting heart rate or moderate/vigorous physical activity. Third, we could not adjust for the influence of medications, especially anti-hypertensive medication.

## Conclusions

In the community-dwelling elderly Chinese, weekly walking activity was significantly associated with a low risk of vascular HMOD (including arterial stiffness, increased CIMT, peripheral artery disease), but not cardiac or renal HMOD. Subgroup analysis demonstrated a significant association between increased daily walking duration and improved vascular HMOD. Hence, increasing daily walking duration (≥1 h/day) would be encouraged in terms of vascular protection.

## Data Availability Statement

The original contributions presented in the study are included in the article/Supplementary Material, further inquiries can be directed to the corresponding author/s.

## Ethics Statement

The studies involving human participants were reviewed and approved by Ethics Committee of Shanghai Tenth People's Hospital. The patients/participants provided their written informed consent to participate in this study.

## Author Contributions

YZ and YX conceived and designed the study. JP put forward many constructive comments for the final version. YL, SY, CC, and JT acquired the data and conducted the statistical analysis. JL, JB, and JP helped data interpretation. YL drafted the manuscript. All authors have read and approved the manuscript.

## Funding

This study was financially supported by the National Nature Science Foundation of China (82170388 and 81800378), Clinical Research Plan of SHDC (SHDC2020CR1040B, SHDC2020CR5009, and SHDC2020CR5015-002), Shanghai Technology Research Leader Program (21XD1434700), and the Cardiac Rehabilitation Fund by the International Medical Exchange Foundation (Z-2019-42-1908-3).

## Conflict of Interest

The authors declare that the research was conducted in the absence of any commercial or financial relationships that could be construed as a potential conflict of interest.

## Publisher's Note

All claims expressed in this article are solely those of the authors and do not necessarily represent those of their affiliated organizations, or those of the publisher, the editors and the reviewers. Any product that may be evaluated in this article, or claim that may be made by its manufacturer, is not guaranteed or endorsed by the publisher.
