# Supplementary material for: Associations of Walking Activity With Hypertensive Mediated Organ Damage in Community-Dwelling Elderly Chinese: The Northern Shanghai Study
Source: Front Cardiovasc Med. 2021 Oct 21;8:734766. doi: 10.3389/fcvm.2021.734766 (PMC8566667; doi:10.3389/fcvm.2021.734766)
Supplement: Supplementary file 1 [file Data_Sheet_1.docx]

Table S1. Correlation of cardiovascular risk factors with walking activity.

| Cardiovascular risk factors | r | *P* |
| --- | --- | --- |
| Gender (male) | 0.011 | 0.54 |
| Age (years) | -0.066 | **<0.001** |
| Smokers | -0.042 | **0.03** |
| Body weight (kg) | -0.10 | 0.59 |
| Body height (cm) | 0.012 | 0.51 |
| Body mass index (kg/m^2^) | -0.018 | 0.34 |
| Systolic blood pressure (mmHg) | -0.032 | 0.09 |

Walking activity was defined as walking more than 10 minutes at a time per week. Pearson correlation analysis was conducted to investigate the correlation of cardiovascular risk factors with walking activity.

Table S2. Subgroup analysis of walking duration with vascular HMOD.

| Vascular HMOD | Non-walking activity  （n=968) | 10-29 min/day  （n=112) | 30-59 min/day  (n=715) | ≥ 1 h/day  (n=1035) | *P* |
| --- | --- | --- | --- | --- | --- |
| Carotid-femoral pulse wave velocity (m/s) | 9.67±2.50 | 9.61±2.36 | 9.46±2.28 | 9.33±2.18^**^ | **0.01** |
| CIMT (µm) | 650.8±162.8 | 631.8±171.8 | 634.4±162.5^*^ | 626.2±145.7^**^ | **0.009** |
| Ankle-brachial index | 1.00±0.14 | 1.01±0.13 | 1.02±0.13^**^ | 1.03±0.11^***^ | **<0.001** |
| Arterial stiffness, n (%) | 147(15.2) | 14(12.5) | 95(13.3) | 114(11.0)^**^ | **0.03** |
| Increased CIMT, n (%) | 71(7.3) | 6(5.4) | 48(6.7) | 43(4.2)^**^ | **0.02** |
| Arterial plaque, n (%) | 614(63.4) | 67(59.8) | 438(61.2) | 661(63.9) | 0.65 |
| Peripheral artery disease, n (%) | 146(15.1) | 14(12.5) | 89(12.4) | 109(10.5)^**^ | **0.01** |

^*^*P*<0.05,^**^*P*<0.01,^***^*P*<0.001 vs Non-walking activity group. DUNCAN’s multiple range tests were conducted to investigate the association of walking duration with vascular HMOD. Abbreviations: CIMT, carotid intima-media thickness; HMOD, hypertensive mediated organ damage.

Table S3. Subgroup analysis of walking frequency with vascular HMOD.

| Prevalence of vascular HMOD (n) | < 3 days/week | ≥ 3 days/week | *P* |
| --- | --- | --- | --- |

| Arterial stiffness  (n=2723) | Yes | 159 | 211 | **0.04** |
| --- | --- | --- | --- | --- |
|  | No | 880 | 1473 |  |
| Increased CIMT  (n=2810) | Yes | 81 | 87 | **0.008** |
|  | No | 1003 | 1639 |  |
| Arterial plaque  (n=2807) | Yes | 686 | 1094 | 0.93 |
|  | No | 394 | 633 |  |
| Peripheral artery disease  (n=2663) | Yes | 160 | 198 | **0.007** |
|  | No | 858 | 1447 |  |

*Chi*-squared tests were conducted to investigate the association of walking days per week with vascular HMOD. Abbreviations: CIMT, carotid intima-media thickness; HMOD, hypertensive mediated organ damage.

Table S4. Subgroup analysis of walking frequency with vascular HMOD using different cut-off walking duration.

| Prevalence of HMOD (n) | | Walking duration ≥ 30 min/day  (n=1750) | | *P* | Walking duration ≥ 1 h/day (n=1035) | | *P* |
| --- | --- | --- | --- | --- | --- | --- | --- |
|  |  | < 3 days/week | ≥ 3 days/week |  | < 3 days/week | ≥ 3 days/week |  |
| Arterial stiffness  (n=2723) | Yes | 165 | 205 | **0.03** | 263 | 107 | **0.004** |
|  | No | 913 | 1440 |  | 1492 | 861 |  |
| Increased CIMT  (n=2810) | Yes | 88 | 80 | **0.001** | 126 | 42 | **0.001** |
|  | No | 1054 | 1588 |  | 1656 | 986 |  |
| Arterial plaque  (n=2807) | Yes | 714 | 1066 | 0.92 | 1142 | 638 | 0.75 |
|  | No | 414 | 613 |  | 665 | 362 |  |
| Peripheral artery disease  (n=2663) | Yes | 166 | 192 | **0.006** | 256 | 102 | **0.003** |
|  | No | 894 | 1411 |  | 1458 | 844 |  |

*Chi*-squared tests wereperformed to investigate the association of walking days per week with vascular HMOD using different cut-off walking duration (≥ 30 min/day and ≥ 1 h/day). Abbreviations: CIMT, carotid intima-media thickness; HMOD, hypertensive mediated organ damage.
